# Median Arcuate Ligament Syndrome in Children: A Single-Center Experience

**DOI:** 10.7759/cureus.57184

**Published:** 2024-03-29

**Authors:** Bahram Kakavand, Robert C Burns, Aliya Centner, Adela Casas-Melley

**Affiliations:** 1 Cardiology, Nemours Children's Health, Orlando, USA; 2 Surgery, Riley Children's Health, Indianapolis, USA; 3 Medicine, University of Central Florida College of Medicine, Orlando, USA; 4 Pediatric Surgery, Nemours Children's Health, Orlando, USA

**Keywords:** postural orthostatic tachycardia syndrome (pots), joint hypermobility, orthostatic intolerance, mals, median arcuate ligament syndrome

## Abstract

Background: Data on median arcuate ligament syndrome (MALS) in children are scant. It is postulated that MALS can cause chronic abdominal pain. It is unclear what percentage of children with this condition are symptomatic and what comorbidities are associated with this syndrome.

Methods: In this retrospective study, data on consecutive patients in a single center diagnosed coincidentally with MALS during routine echocardiogram were reviewed. Symptom burden, comorbidities, and the effect of anthropometric indices on MALS were investigated. Descriptive statistics and nonparametric tests were used to describe the findings and to compare variables with normal distribution.

Results: Between 2013 and 2020, there were 82 children, 55 females (67%), mean age 13.9 ± 3.2 years, with MALS and complete record. Mean velocity across the stenotic area was 2.6 ± 0.4 m/s. Forty-six patients (57%) had abdominal pain. Age, gender, weight, body mass index (BMI), and Doppler velocity had no statistically significant influence on symptom occurrence. Conversely, patients with joint hypermobility and symptoms of orthostatic intolerance were more likely to have abdominal pain from MALS. Of 24 patients with joint hypermobility, 18 patients had abdominal pain (p=0.027). Thirty-eight patients with orthostatic intolerance (OI) with MALS complained of abdominal pain vs 13 patients with OI and no abdominal pain (p=<0.0001).

Conclusion: Nearly half of patients with MALS had abdominal pain. Age, gender, weight, and the degree of stenosis had no statistically significant influence on symptom occurrence. OI, specifically postural orthostatic tachycardia syndrome (POTS), and joint hypermobility on exam predicted a higher propensity for abdominal pain in patients with MALS.

## Introduction

Median arcuate ligament syndrome (MALS), first reported by Harjola in 1963 [1] and also known as celiac artery compression syndrome or Dunbar syndrome [2], is considered a rare condition. MALS is characterized by the compression of the celiac artery due to inferior malposition of the median arcuate ligament (MAL). MAL is formed by the crura diaphragmatica and builds the anterior base of the ventral aortic hiatus of the diaphragm. Under normal circumstances, the ligament lies superior to the celiac artery at the level L1 vertebral body. At times, the ligament is displaced inferiorly and crosses over the base of the celiac artery. This gives a hook-shaped impression of the artery in computer tomography angiography (CTA) or magnetic resonance angiography (MRA) imaging.

Typically, in inspiration, the celiac artery is displaced inferiorly and away from the median arcuate ligament. This explains the observation during computer tomography or magnetic resonance angiogram that the stenosis is relieved in inspiration and further worsens during expiration.

Accurate data on MALS prevalence are not available given that many individuals with MALS have no symptoms, and systemic, population-based surveillance is lacking. Petnys et al. reported their observation on the prevalence of celiac axis compression on computed tomography angiography in asymptomatic patients [3]. Celiac artery compression occurred in 3.4% of the normal asymptomatic population [3]. In another study from 1971, MALS incidence in the adult population was estimated to be 10%-24% [4]. The pathophysiology of MALS remains unclear. While many affected individuals have no relevant symptoms, others complain of chronic abdominal pain of varying degrees. It is believed that the ischemia to foregut is the etiology of the pain. Collateral formation may alleviate the ischemia and symptoms. Another theory points to a steal phenomenon from other arteries causing ischemia [5].

The diagnosis of MALS is made by sonography. From a subcostal sagittal view, the abdominal aorta and its branches are visualized. At times, Doppler evaluation of the celiac artery from sagittal view becomes inaccurate due to a large angle between the celiac artery axis and the Doppler. In this case, a subcostal coronal view can visualize the aorta in cross-section with an anterior take-off of the celiac artery perpendicular to the Doppler incident angle. A definitive diagnosis is made with advanced imaging techniques like CTA or MRA.

Data on MALS in children are scant. Mak et al. reported 46 patients under the age of 21 years with MALS. Ninety-one percent were female [6]. All patients underwent successful surgical release. Eight of nine patients with complications required secondary reintervention [6]. In 2006, Scholbach published a German center’s experience with children with MALS. They concluded that MALS is more prevalent than expected. Abdominal pain was observed in 71% of the population [7]. In this retrospective study, we report on pediatric MALS patients’ characteristics with respect to demographics and symptom burden. This article was previously presented as a virtual poster at the 2020 American Autonomic Society Annual Meeting on November 6-7, 2020.

## Materials and methods

**Figure 1:**
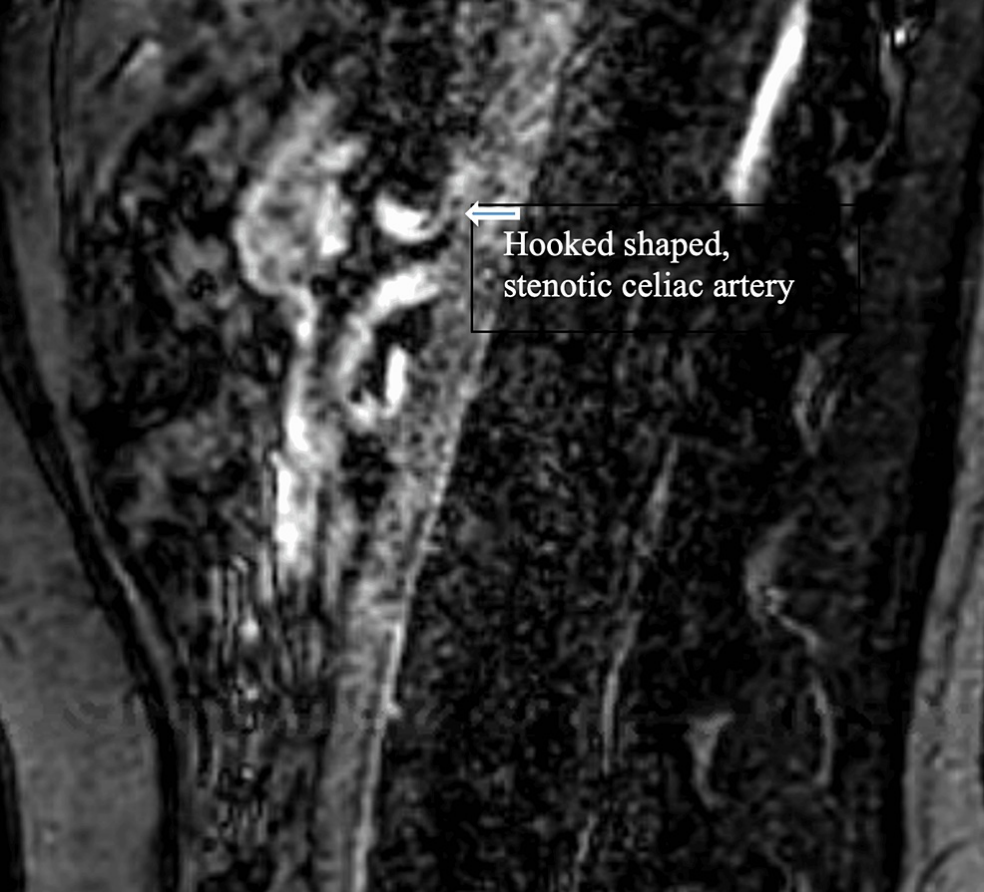
MRA of Celiac Artery Stenosis Magnetic resonance angiography (MRA) shows take-off of celiac artery with severe stenosis hook-shaped deformity (arrow).

In this retrospective study, electronic charts of patients visiting the cardiology outpatient clinic at Nemours Children’s Hospital in Orlando, FL, between November 2013 and April 2020 with the diagnosis of median arcuate ligament syndrome or celiac artery stenosis were reviewed. A total of 82 patients under the age of 19 years were found to have adequately and accurately recorded data. All patients had undergone an echocardiogram. The author’s (BK) imaging protocol calls for routine Doppler evaluation of the celiac artery from the subcostal view in all patients, assuming adequate image quality and child’s cooperation. The inclusion of celiac artery Doppler evaluation began after the author’s encounter with a patient with celiac artery stenosis and chronic abdominal pain in 2012. Depending on the clinical circumstances, some patients underwent advanced confirmatory imaging like MRA (Figure 1) or CTA. The preferred technique has been MRA. In some cases, a CTA was dictated by the patient’s insurance. This study was approved by the local institutional review board.

Echocardiographic imaging

**Figure 2:**
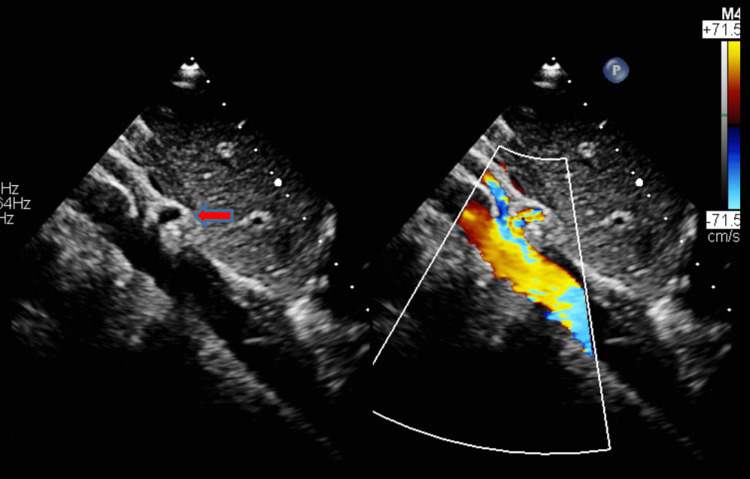
Two-Dimensional and Color Doppler of the Abdominal Aorta in Subcostal Sagittal View The diagnosis of MALS can be made by sonography. From a subcostal sagittal view, the abdominal aorta and its branches are visualized. The red arrow points to celiac artery with a stenotic base and poststenotic dilation. MALS: median arcuate ligament syndrome.

**Figure 3:**
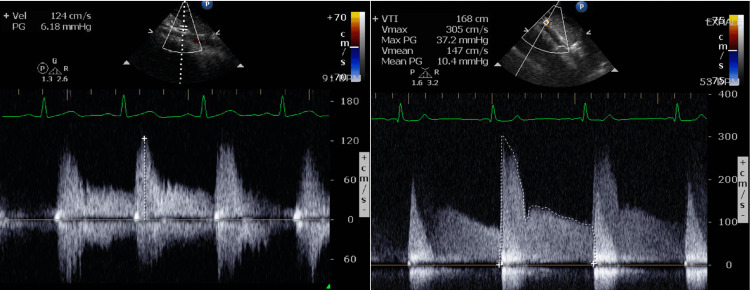
Celiac Artery Doppler On the left, a low-velocity pulsed Doppler of a normal celiac artery is shown. On the right, there is a high-velocity flow with significant diastolic run-off. The angle between the Doppler and celiac artery axis is very small.

The abdominal aorta is visualized from the subcostal sagittal view in two-dimensional and color Doppler (Figure 2). The Doppler interrogation is begun with a pulsed wave. In case of aliasing, continuous Doppler is used (Figure 3). A velocity ≥2 m/s is considered abnormal [8].

**Figure 4:**
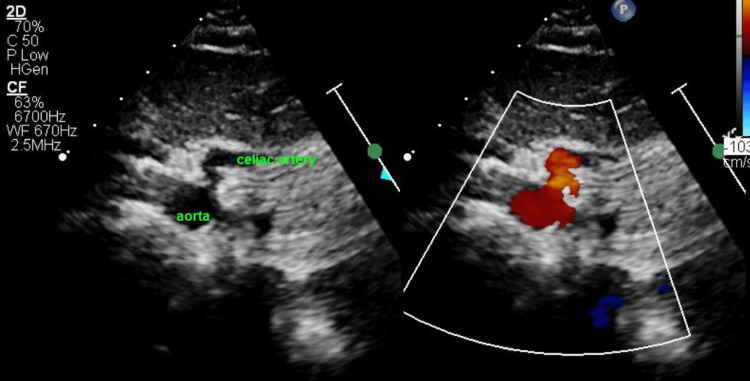
Subcostal Coronal View of the Aorta In subcostal coronal view, the aorta is shown in cross-section with celiac artery arising almost perpendicularly. This allows accurate Doppler interrogation of the vessel.

If the Doppler angle of incidence is greater than 30 degrees compared to the celiac axis, aorta and celiac artery are imaged from the subcostal coronal view (Figure 4). This adjustment provides adequate Doppler interrogation. Inspiratory and expiratory gradient was attempted initially in some patients. The image quality decay with inspiration led us to abandon this technique.

Surgical technique

**Figure 5:**
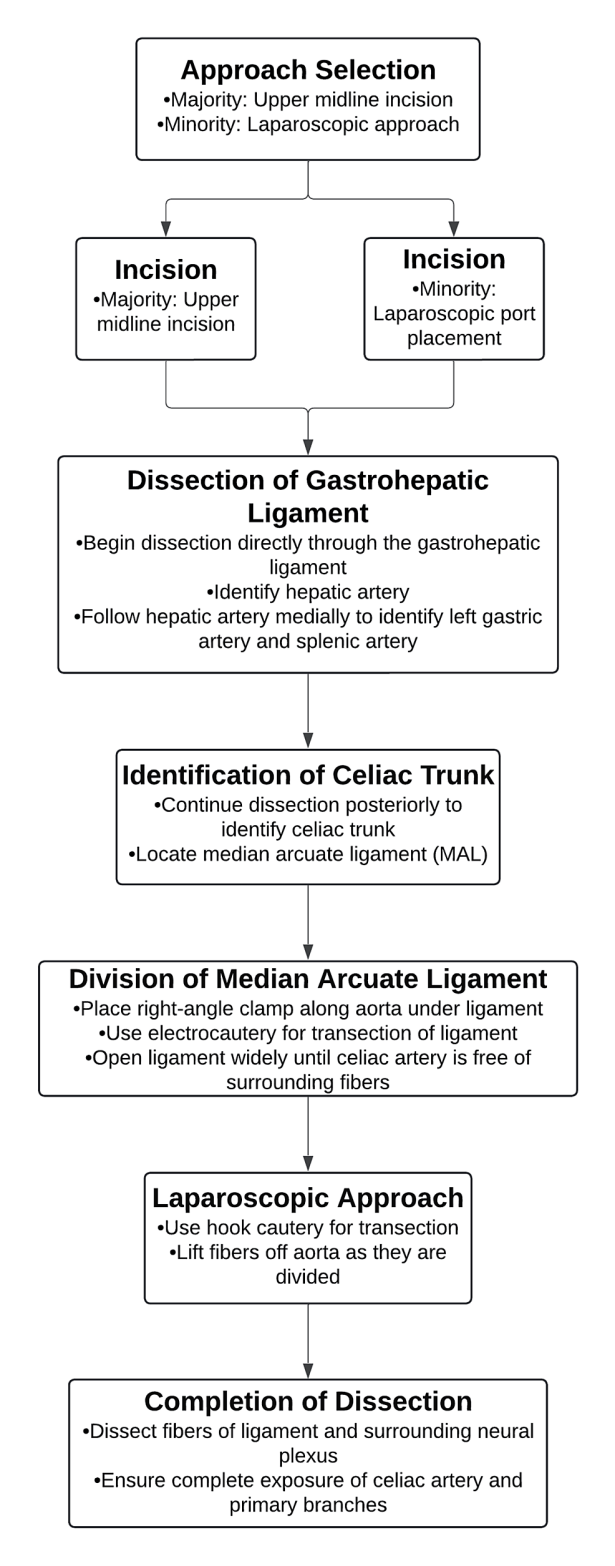
Schema of MALS Surgical Technique The surgical technique used to alleviate the symptoms of median arcuate ligament syndrome (MALS) is detailed.

Most patients were approached through an upper midline incision, although a few did undergo a laparoscopic approach. Both approaches were done similarly. Dissection was begun directly through the gastrohepatic ligament. This exposure allowed the identification of the hepatic artery. The hepatic artery was then followed medially with identification of the left gastric artery and the splenic artery. Following these vessels posteriorly allowed for the identification of the celiac trunk which led to the median arcuate ligament. Dissection proceeded in a caudal direction on the anterior wall of the aorta. Division of the fibers of the median arcuate ligament (MAL) was best accomplished by placing a right-angle clamp along the aorta under the ligament, and then electrocautery was used to transect the ligament. The ligament was opened widely until the celiac artery was completely free of surrounding fibers. Laparoscopically, the hook cautery was used for transection, which allows one to lift the fibers off the aorta as they are divided. Dissection of the fibers of the ligament as well as any surrounding neural plexus was completed until the celiac artery and the primary branches were completely exposed. This surgical technique is detailed in Figure 5.

Management of MALS

Asymptomatic patients were not further pursued for this finding, although parents were made aware of it. Patients with MALS and abdominal pain and negative prior gastrointestinal (GI) workup who asked for advice were referred to pediatric surgery for consultation and management. The remainder of the patients were referred back to their primary care team.

Statistics

Descriptive statistics were used to describe patients’ demographics and clinical characteristics. Nonparametric test (Chi-square) was employed to analyze the group difference within a variable or to compare observations with non-Gaussian distribution. The analysis was performed on SPSS, Version 25 (IBM Corp. Released 2017. IBM SPSS Statistics for Windows, Version 25.0. Armonk, NY: IBM Corp).

## Results

Eighty-two patient charts were available for review with complete records, who were seen over a period of six and half years (2013-2020). Fifty-five patients (67%) were female. The average age at the time of diagnosis was 13.9 ± 3.2 years (range: 3-17 years, median: 15 years). Average weight was 50.4 ± 17.2 kg (range: 13.4-106 kg, median: 54.1 kg). Most patients had normal weight (average BMI: 20.6 ± 4.2 kg/m^2^). Only nine patients or 10.9% were overweight or obese. These variables are further analyzed based on gender in Table 1.

**Table 1:** Patient Characteristics There was no statistical significance between genders for any given variable. M: male; F: female; BMI: body mass index; n: case number; SD: standard deviation.

	Gender	n	Mean	SD
Age	M	27	12.6	4.0
	F	55	14.5	2.6
Weight	M	27	53.2	23.7
	F	55	54.5	13.3
BMI	M	25	19.7	5.0
	F	53	21.1	3.7

Patients’ referral symptoms/diagnoses to our cardiology clinic were variable. They were divided into orthostatic intolerance (OI) related including dizziness, syncope, and postural orthostatic tachycardia syndrome (POTS) and OI unrelated including chest pain, congenital heart disease, palpitation, murmur, abnormal electrocardiogram, bradycardia, hypertension, and tachyarrhythmias. OI-related referrals were 51 cases (62%, p=0.027), of which 20 patients were later diagnosed with POTS.

**Figure 6:**
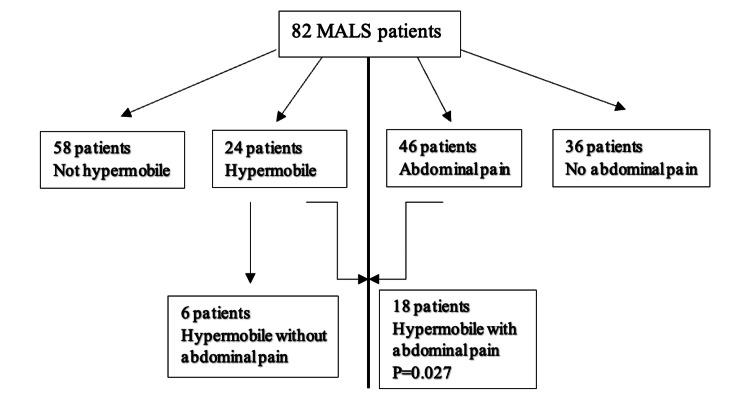
MALS Patients and Joint Hypermobility The patients are divided based on the presence or absence of joint hypermobility (left half) and abdominal pain (right half). Patients with median arcuate ligament syndrome (MALS) and joint hypermobility (hypermobile) were more likely to have abdominal pain.

MALS can be associated with gastrointestinal symptoms. In our study, 46 patients (56%) had abdominal pain. Pain was described as constant, postprandial, postexertional, or any combination of the three pain types. Of the 82 patients with MALS, 24 patients (29.3%) demonstrated joint hypermobility of varying severity. When patients with abdominal pain were analyzed for joint hypermobility, 18 patients with joint hypermobility were symptomatic with pain vs six hypermobile patients without pain (p=0.027; Figure 6).

Patients with OI-related referral symptoms and MALS were highly likely to have abdominal pain (p≤0.0001). More specifically, patients with POTS and MALS were more likely to have pain as compared with non-POTS patients (p=0.014; Table 2).

**Table 2:** MALS-Related Abdominal Pain Association With OI, POTS, and Joint Hypermobility This table shows the distribution of patients with median arcuate ligament syndrome (MALS)-related abdominal pain and orthostatic intolerance (OI) symptoms, postural orthostatic tachycardia syndrome (POTS), and joint hypermobility (Hypermobile). “+” depicts symptom present and “–“ symptom not present.

	OI	POTS	Hypermobile
Abdominal pain -	13	4	6
Abdominal pain +	38	16	18
p value	<0.0001	0.014	0.027

On echocardiogram, the flow velocity in the stenotic celiac artery was 2.6 ± 0.4 m/s. There was no difference in the velocity between male and female patients. Age and gender also had no influence on developing abdominal pain (p values=0.39 and 0.14, respectively).

Management of MALS

Of 46 patients with MALS and abdominal pain, 26 patients underwent CTA or MRA to confirm the diagnosis in advance of a possible surgical intervention. Sixteen patients ultimately decided to undergo surgery after lengthy conversation with authors (BK, RCB, and ACM) including four patients who were referred to other centers due to a gap between in-house pediatric surgeons. These patients had had prior negative gastrointestinal workup. The indication for surgery was a constellation of confirmed MALS by CTA or MRA and the presence of abdominal pain with a negative prior gastrointestinal workup. All patients experienced complete or near-complete resolution of pain. In one patient, the pain recurred after 10 days. The repeat ultrasound shortly after surgery had shown normal flow velocity in the celiac artery. When abdominal pain recurred, the ultrasound revealed restenosis. A second intervention was performed resulting in symptom resolution. In general, a follow-up sonographic evaluation of the celiac artery was not obtained after repair, if the patient became symptom free. Of 16 patients who underwent surgical repair, only five patients are still seen in our clinic for their orthostatic complaints. Abdominal pain has not recurred. One male patient, now 17 years old, returned after six years with a recurrence of abdominal pain and dizziness. The patient was referred to the gastroenterology division for a complete workup.

## Discussion

We studied the characteristics of an unselected patient population in our cardiology clinic with MALS as a coincidental finding. MALS is an under-recognized cause of chronic abdominal pain. While the pathophysiological changes with respect to the median arcuate ligament are well understood, the actual cause of abdominal pain (or lack of pain in some patients) is less well understood. Downstream ischemia and steal phenomenon are among the considerations. The role of arterial collaterals is not clearly known. The published information on the prevalence of MALS varies widely between studies. Scholbach cited a prevalence of 1.7% in the pediatric population [7]. Petnys et al. reported a prevalence of 3.4% in CT angiographic studies in asymptomatic adult patients [3]. These statistics are not contradictory; these studies are designed differently and reached different conclusions.

To our knowledge, this retrospective review represents the largest pediatric population with MALS. Our study included two very young patients, three and seven years old at the time of diagnosis. Scholbach reported a 22-month-old toddler with this condition [7]. It is not clear what process leads to increasing impingement of the celiac artery by the arcuate ligament with age. Our study differs from other publications given that MALS was a coincidental finding during echocardiography, and our aim was not to diagnose the etiology of abdominal pain. This allowed us to have an unselected patient population.

One important finding of this study is that MALS does not cause abdominal pain in all affected patients. In fact, 36 patients (43%) did not have any GI symptoms. Factors that were statistically unimpactful on developing abdominal pain included age, gender, weight, and Doppler velocity in the stenotic celiac artery. The latter finding contradicts previous reports that symptoms were likely to occur with a higher degree of stenosis [9].

Patients’ initial presentation and referral symptom did correlate with abdominal symptoms. Patients who were referred to our cardiology clinic for evaluation of orthostatic intolerance symptoms (dizziness, syncope, POTS) were more likely to have abdominal pain associated with MALS (53 patients, p≤0.0001). After undergoing further workup for OI, 20 patients were diagnosed with POTS. Patients with POTS and MALS were more likely to have pain as compared with non-POTS patients (p=0.014). We previously reported our experience with MALS and POTS [10]. In that study, we found that the incidence of MALS was higher in pediatric POTS patients than those without POTS. We recommended at the time that POTS patients with abdominal symptoms be screened for celiac artery stenosis.

Another aspect of the present study is the finding of a higher incidence of symptomatic MALS in patients with joint hypermobility. In general, only 24 patients (29.3%) with MALS had joint hypermobility. Conversely, however, 18 of the 24 patients (75%) had abdominal pain. Other studies have similarly found an increased prevalence of Ehler-Danlos Syndrome and POTS in patients with MALS [11].

It remains unclear why patients with OI symptoms and joint hypermobility have a higher incidence of MALS and abdominal pain. Factors under consideration are 1) the confounding effect of venous pooling in the splanchnic system and 2) lower perfusion pressure in orthostatic patients in an already impaired circulation in the celiac artery perfusion area.

This study also demonstrates the success rate of MALS surgery as 16 patients underwent surgery with only one requiring reintervention for recurring abdominal pain. The rest of the patients achieved either complete alleviation or near-total relief from their pain. While not performed in these patients, endovascular stent placement may also be used as a treatment technique. However, it does have high failure rates and is largely used as a secondary intervention following the failure of the initial surgical technique [12].

There are some limitations in this retrospective study. Firstly, the study was conducted at a single center, which may limit the generalizability of the findings to other populations. Additionally, the study population consisted of patients referred to a cardiology clinic, which might introduce selection bias as patients with more severe symptoms or comorbidities could have been more likely to be referred. Long-term follow-up data for all patients would be helpful in order to assess the durability of symptom relief after surgical intervention and to identify potential late complications or recurrences. Despite these limitations, this study provides valuable insights into the characteristics and clinical presentation of pediatric patients with MALS, highlighting the need for further research to better understand the pathophysiology and optimal management of this rare condition.

## Conclusions

MALS is seen infrequently in children. Nearly half of the patients had no abdominal symptoms. Patients with orthostatic intolerance symptoms and joint hypermobility are more likely to have abdominal pain from MALS. Age, gender, weight, or the degree of celiac artery stenosis had no influence on the occurrence of pain in MALS. Differential diagnosis of unclear abdominal pain particularly in children with orthostatic intolerance symptoms should include MALS.
